# MNX1-AS1 suppresses chemosensitivity by activating the PI3K/AKT pathway in breast cancer

**DOI:** 10.7150/ijbs.104483

**Published:** 2025-05-27

**Authors:** You Shuai, Zhonghua Ma, Jian Yue, Chunxiao Li, Jie Ju, Xue Wang, Haili Qian, Peng Yuan

**Affiliations:** 1 Department of Medical Oncology, National Cancer Center/National Clinical Research Center for Cancer/Cancer Hospital, Chinese Academy of Medical Sciences and Peking Union Medical College, Beijing, 100021, China.; 2 Key Laboratory of Carcinogenesis and Translational Research (Ministry of Education), Department of Endoscopy, Peking University Cancer Hospital & Institute, Beijing, 100142, China.; 3 Department of VIP Medical Services, National Cancer Center/National Clinical Research Center for Cancer/Cancer Hospital, Chinese Academy of Medical Sciences and Peking Union Medical College, Beijing, 100021, China.; 4 State Key Laboratory of Molecular Oncology, National Cancer Center/National Clinical Research Center for Cancer/Cancer Hospital, Chinese Academy of Medical Sciences and Peking Union Medical College, Beijing, 100021, China.

**Keywords:** LncRNA, breast cancer, chemosensitivity, ITGA6/PI3K/AKT pathway, lipid nanoparticles

## Abstract

Long noncoding RNAs (lncRNAs) critically regulate tumorigenesis and chemosensitivity. Despite the pivotal role of lncRNAs in breast cancer (BC), their specific functions and underlying mechanism, particularly in the context of drug resistance, remain largely unexplored. We discovered that MNX1-AS1 is significantly elevated in BC and contributes to paclitaxel resistance through the PI3K/AKT pathway. Moreover, elevated MNX1-AS1 expression exhibits close association with unfavourable prognosis in BC. Mechanistically, MNX1-AS1 interacts with YBX1, preventing its SMURF2-mediated ubiquitination and subsequent degradation, thereby increasing YBX1 protein levels. Upregulated YBX1 transcriptionally activates the expression of ITGA6 by binding to its promoter in the nucleus. Furthermore, MNX1-AS1 binds to IGF2BP2, promoting the stability of ITGA6 mRNA in an m6A-dependent manner within the cytoplasm. MNX1-AS1 increases ITGA6 expression at transcriptional and post-transcriptional levels, thereby activating the PI3K/AKT pathway. Notably, lipid nanoparticles were implicated to effectively deliver MNX1-AS1 siRNA to tumor-bearing mice, resulting in significant antitumor effects. These findings underscore the role of MNX1-AS1 in activating the ITGA6/PI3K/AKT pathway, which facilitates tumor progression and induces chemoresistance in BC. Targeting MNX1-AS1 may represent a promosing therapeutic strategy to enhance chemotherapy efficacy in BC patients.

## Introduction

According to the Global Cancer Statistics 2020 report, breast cancer (BC) is the most commonly diagnosed malignancy among women and ranks as the leading cause of cancer-related deaths worldwide^1^. The clinical management of diverse BC types encompasses a range of therapeutic strategies, including endocrine therapy^2^, HER2-targeted therapy^3^, chemotherapy^4^, and immunotherapy^5^. Although chemotherapy remains a cornerstone treatment for BC and significantly improves patient prognosis, the varying sensitivity of BC cells to chemotherapeutic agents plays a crucial role in determining treatment outcomes^4^. The modulation of drug resistance is governed by the complex interplay of multiple factors and pathways that govern drug sensitivity. However, the intricacies of this regulatory network remain poorly understood. Therefore, a comprehensive investigation into the molecular mechanisms underlying chemotherapy resistance in BC is imperative to refine and guide the formulation of comprehensive treatment strategies.

Long noncoding RNAs (lncRNAs) are key regulators of cellular functions and significantly impact tumor behaviour and pathology by modulating genes critical for cancer growth and progression^6^-^9^. Our previous studies have demonstrated that lncRNA MNX1-AS1, which is transcriptionally activated by the TEA domain family member 4 (TEAD4), is closely linked to the progression of gastric cancer (GC)^10^. Mechanistically, MNX1-AS1 exerts its effects through the EZH2/BTG2 and miR-6785-5p/BCL2 pathways in GC^11^. Additionally, the involvement of MNX1-AS1 has been documented in various cancers, including lung cancer^12^, cholangiocarcinoma^13^, and gallbladder cancer^10^. However, the diverse mechanisms by which MNX1-AS1 contributes to aberrant cellular behaviour in BC remain largely unexplored. Notably, lncRNAs play crucial roles in driving drug resistance, posing a major obstacle to effective cancer treatment. For instance, lncRNA DIO3OS interacts with polypyrimidine tract-binding protein 1 (PTBP1) to upregulate LDHA expression, thereby driving metabolic reprogramming and promoting resistance to aromatase inhibitor in BC 14. Moreover, lncRNA HCP5 induces fatty acid oxidation (FAO)-mediated chemoresistance through the miR-3619-5p/AMPK/PGC1α/CEBPB axis^15^. However, the involvement of MNX1-AS1 in mediating drug resistance in BC remains completely uncharacterized. Further delineating the mechanism of MNX1-AS1 in BC progression and chemosensitivity is crucial for uncovering novel insights into its underlying pathological mechanism and potential therapeutic targets.

Therapeutic targeting of lncRNAs encounters significant hurdles in drug development. However, RNA interference (RNAi) therapy, particularly the use of small interfering RNA (siRNA), has emerged as a promising strategy in cancer treatment owing to its ability to specifically silence disease-associated genes. Lipid nanoparticles (LNPs) efficiently package siRNA and facilitate its delivery into cells for targeted gene silencing^16^,^17^. Moreover, siRNA-based strategies targeting lncRNAs have demonstrated significant clinical potential, highlighting their capacity to precisely modulate gene expression^18^. Consequently, the integration of siRNA-based strategies targeting lncRNAs with lipid nanoparticle technology represents a promising frontier in clinical therapeutics. This synergistic combination not only offers a precise method for modulating lncRNA activity but also holds the potential to revolutionize BC treatment.

The ITGA6 gene encodes the integrin α6 subunit, a crucial component of extracellular matrix receptors that play a vital role in facilitating cell adhesion and signal transduction. Upon forming complexes with ITGB4 (CD104) or ITGB1 (CD29), ITGA6 assembles into either α6β4 integrin or α6β1 integrin, respectively. These integrin complexes are essential for various cellular interactions and signalling pathways^19^,^20^. Aberrant expression of ITGA6 has been implicated in the initiation and progression of various tumors^21^-^23^. ITGA6 activation triggers the phosphoinositide 3-kinase (PI3K)/protein kinase B (AKT) pathway, which regulates cell division, growth, and survival. Dysregulated activation of the PI3K/AKT pathway is a pivotal contributor to chemotherapy resistance, diminishing the effectiveness of anticancer drugs^24^-^26^. For example, AKT overexpression has been shown to confer resistance to apoptosis-inducing chemotherapy drugs, including paclitaxel and doxorubicin, in breast and ovarian cancer cells^27^. Furthermore, mutations in PI3K or PTEN, a tumor suppressor that negatively regulates the PI3K/AKT pathway, have been shown to display close association with increased chemoresistance in various cancers, including prostate and endometrial cancers^28^.

In this study, integrated bioinformatics analysis and clinical tissue sample examinations revealed that MNX1-AS1 expression is significantly upregulated in BC tissues compared to normal controls, and this overexpression correlates strongly with poor clinical outcomes. Furthermore, MNX1-AS1 is implicated in enhancing the expression of ITGA6/PI3K/AKT pathway components in BC cells, thus contributing to malignant progression and paclitaxel resistance in BC. This study, for the first time, highlights the critical role of MNX1-AS1 in modulating drug efficacy in BC. Mechanistically, our findings indicate that MNX1-AS1 directly interacts with Y-box binding protein 1 (YBX1), inhibiting its ubiquitination and subsequent degradation. This inhibition results from MNX1-AS1 competitively disrupting the interaction between SMAD ubiquitination regulatory factor 2 (SMURF2) and YBX1. Consequently, increased YBX1 levels transcriptionally activate ITGA6 expression. Furthermore, MNX1-AS1 interacts with IGF2BP2, facilitating its recruitment to recognize the N6-methyladenosine (m6A) methylation of ITGA6, thereby maintaining ITGA6 stability. In conclusion, our research elucidates the mechanistic role of MNX1-AS1 in driving malignant progression and chemoresistance in BC cells. Furthermore, we propose a novel therapeutic approach utilizing lipid nanoparticles (LNPs) encapsulating MNX1-AS1-specific siRNA, which demonstrates significant therapeutic potential in BC. These observations pave the way for further exploration and development of BC therapeutics.

## Materials and methods

### Human tissue specimens

This study involved two groups of patient samples. Group 1 samples were included in the BC tissue microarray purchased from Shanghai Zhuoli Biotech Co., Ltd. (Shanghai, China). The samples included tumor tissues and paired breast tissues from 90 surgically treated BC patients. Group 2 samples were obtained from the pathology department of the Chinese Academy of Medical Sciences and Peking Union Medical College (CHCAMS) and consisted of paraffin-embedded specimens of tumor tissue from 17 BC patients who underwent surgical treatment and received the usual postoperative treatment. All the participants who provided tissue samples signed a written informed consent form. The use of these clinical specimens was authorized by the Institutional Research Ethics Committee of the Cancer Hospital at the Chinese Academy of Medical Sciences.

### Cell line culture

The BC cell lines MCF-7 and MDA-MB-231, which were obtained from the American Type Culture Collection (ATCC, Manassas, VA, USA), were maintained in culture. These cell lines were grown in Dulbecco's modified Eagle's medium (DMEM, HyClone, Logan, UT, USA) supplemented with 10% foetal bovine serum (FBS, HyClone, Logan, UT, USA) and a 1% penicillin‒streptomycin mixture. Cells were cultured under standard conditions in a humidified incubator maintained at 37°C with 5% CO_2_.

### RNA extraction and qRT‒PCR assays

Total RNA was extracted with TRIzol reagent (Invitrogen, Grand Island, NY, USA) following the manufacturer's instructions. Quantitative real-time PCR (qRT‒PCR) was carried out following the manufacturer's instructions (Takara, Dalian, China). Details concerning the utilized primers are listed in Table S2.

### Cell transfection

The details of the cell transfection assay are described in the Supplementary Materials.

### EdU assay

An EdU labelling/detection kit (RiboBio, Guangzhou, China) was used for the EdU assays. For detailed information, refer to the Supplementary Materials and Methods.

### Cell proliferation assay

Cell proliferation was measured using an MTS assay. The cells were plated at an initial density of 3×10^3^ cells per well in 96-well plates. For the assay, at predetermined time points, 10 μl of MTS reagent was added to each well containing 100 μl of cell culture in the 96-well plates. The plates were then returned to the incubator and incubated at 37°C for 1 to 3 hours. The optical density (OD) was measured at 490 nm using a microplate reader.

### Western blotting

Western blotting was performed as previously reported^11^. All the antibodies used are listed in Table S2.

### Xenotransplantation mouse model and treatment

All animal experiments adhered to protocols approved by the Animal Experiment Ethics Committee of the Chinese Academy of Medical Sciences Cancer Hospital (CHCAMS). Female BALB/c nude mice, aged 4-5 weeks, were acquired from HFK Biotechnology (Beijing, China) and maintained in a sterile, controlled environment with a consistent diet. We used a calliper to measure the tumor dimensions and calculated the volume of the mouse tumors as follows: (length × width ^ 2)/2.

### Paclitaxel-resistant mouse model

MDA-MB-231 (3×10^6^) cells with or without MNX1-AS1 knockout were subcutaneously injected into the right side of the abdomen of the mice. The tumor-bearing mice were stratified on the basis of their initial tumor volume, and within each group, the mice were randomly assigned to the DMSO control group or the paclitaxel treatment group using a computer-generated random number table. After grouping, the baseline tumor volume and body weight were verified to ensure that there were no significant differences between the groups. An independent investigator prepared the paclitaxel and DMSO to ensure that the appearance and injection volume of the solutions were identical. Drug injections and tumor measurements were carried out by researchers who were blinded to the group assignments to minimize potential bias. DMSO or paclitaxel (5 mg/kg) was administered intraperitoneally every 2 days, for a total of six injections. The tumor dimensions were evaluated every 2 days.

### MNX1-AS1-depleted mouse model

MDA-MB-231 (3×10^6^) cells were subcutaneously inoculated into the right flanks of 4-week-old BALB/c-nude mice. Tumor-bearing mice were stratified by initial tumor volume and randomly assigned to the LNP-si-MNX1-AS1 or LNP-si-CTRL group using a computer-generated random number table. The baseline tumor volume and body weight were checked to ensure that there were no significant differences between the groups. An independent person encoded the treatments to match the appearance and injection volume of the solutions. Drug injections and tumor measurements were performed by blinded researchers to minimize bias. The mice were treated with LNP-si-MNX1-AS1 (5 µg/mouse) or LNP-si-CTRL (5 µg/mouse) every 3 days. All the mice were euthanized after 12 days of treatment.

### Isolation of cytoplasmic and nuclear RNA

Cytoplasmic and nuclear RNAs were extracted and purified following previously established methods^11^.

### Immunohistochemistry (IHC) analysis

Breast tumor samples were processed for immunohistochemistry, the signal was enhanced with 3,3′-diaminobenzidine, and the samples were counterstained with haematoxylin. Positive expression was defined as staining in 50% or more of the cancer cells. Protein expression levels via evaluation of both the intensity and extent of staining (performed by two independent pathologists unaware of the patient details). The intensity of staining was graded from 0 (no staining) to 3 (strong staining), and the extent of staining was graded from 0 (less than 25% cells) to 3 (75% to 100% cells). The overall score, ranging from 0 to 9, was obtained by multiplying the staining intensity and extent scores.

### RNA sequencing

RNA sequencing was carried out as previously described^11^.

### Coimmunoprecipitation (Co-IP)

To evaluate the physical interactions between proteins, MDA-MB-231 and MCF-7 cells were lysed in NP-40 lysis buffer. A total of 1 mg of cell lysate was incubated with 2 µg of YBX1 antibody or 2 µg of SMURF2 antibody overnight on a rotating shaker at 4°C, and then 20 µL of Protein A/G beads (sc2003, Santa Cruz) was incubated at 4°C for 6 hours. The immunoprecipitate was washed six times and collected after centrifugation at 3500 x g for 5 minutes. The beads were subsequently boiled in SDS‒PAGE buffer for 10 minutes, after which protein blotting was performed. All the experiments were conducted in triplicate.

### RNA immunoprecipitation (RIP)

RIP experiments were conducted using established protocols^11^. Detailed information about the antibodies used is available in Table S2.

### Chromatin immunoprecipitation (ChIP) assays

The ChIP experiments were carried out in accordance with previously described methods^11^. The primers used are specified in Table S2.

### Luciferase assays

Luciferase assays were conducted using a luciferase assay kit (Promega, Madison, WI, USA) following the manufacturer's instructions. YBX1 binding sites on the ITGA6 promoter were identified using JASPAR, and various sequences were subsequently cloned and inserted into a pGL4.10 vector for assay evaluation.

### Fluorescence in situ hybridization (FISH) and subcellular separation

The FISH and subcellular separation experiments were executed as previously described^11^. The probe sequences are detailed in Table S2.

### Colony Formation Assay

Colony formation experiments were conducted as previously reported^11^.

### Generation of siRNA LNPs

The preparation of LNP-si-CTRL and LNP-si-MNX1-AS1 was carried out using a microfluidic mixing method, which allowed the lipid and mRNA mixture to fully and rapidly form LNPs with uniform particle sizes. In brief, a volume of lipid-ethanol mixture (comprising D-Lin-MC3, DSPC, cholesterol, and DMG-PEG2000) and three volumes of mRNA-citrate buffer were loaded into the microfluidic chip for mixing. The resulting LNP-siRNA suspension was subsequently diluted in citric acid buffer and subjected to ultrafiltration. Before being added to the cell cultures, the LNP-siRNA suspension was further diluted in PBS or culture medium to reduce the trace amounts of ethanol to nontoxic levels (<0.5%).

### RNA stability assays

The details of the RNA stability assays are described in the supplementary material.

### Cycloheximide (CHX) chase experiment

Following treatment with 50 μg/ml CHX (MedChemExpress, USA), the cells were harvested at 0, 3, 6, 9 and 12 h to extract proteins for western blot analysis.

### Methylated RNA immunoprecipitation (MeRIP) assay

Total RNA was isolated from MDA-MB-231 cells, and mRNA was broken into fragments of approximately 300 nt. Anti-N6 methyladenosine or IgG antibodies were used for the immunoprecipitation reaction, followed by washing with RIP washing solution. Finally, RNA was extracted via the phenol/chloroform method.

### Statistical analysis

Statistical evaluations were performed using SPSS 17.0 software (IBM, USA). Differences between groups were assessed using a paired, two-tailed Student's t test, χ2 test, or Wilcoxon test, depending on suitability. P values less than 0.05 were considered to indicate statistical significance.

## Results

### MNX1-AS1 is significantly upregulated in BC and correlates with adverse prognostic outcomes

**Figure 1:**
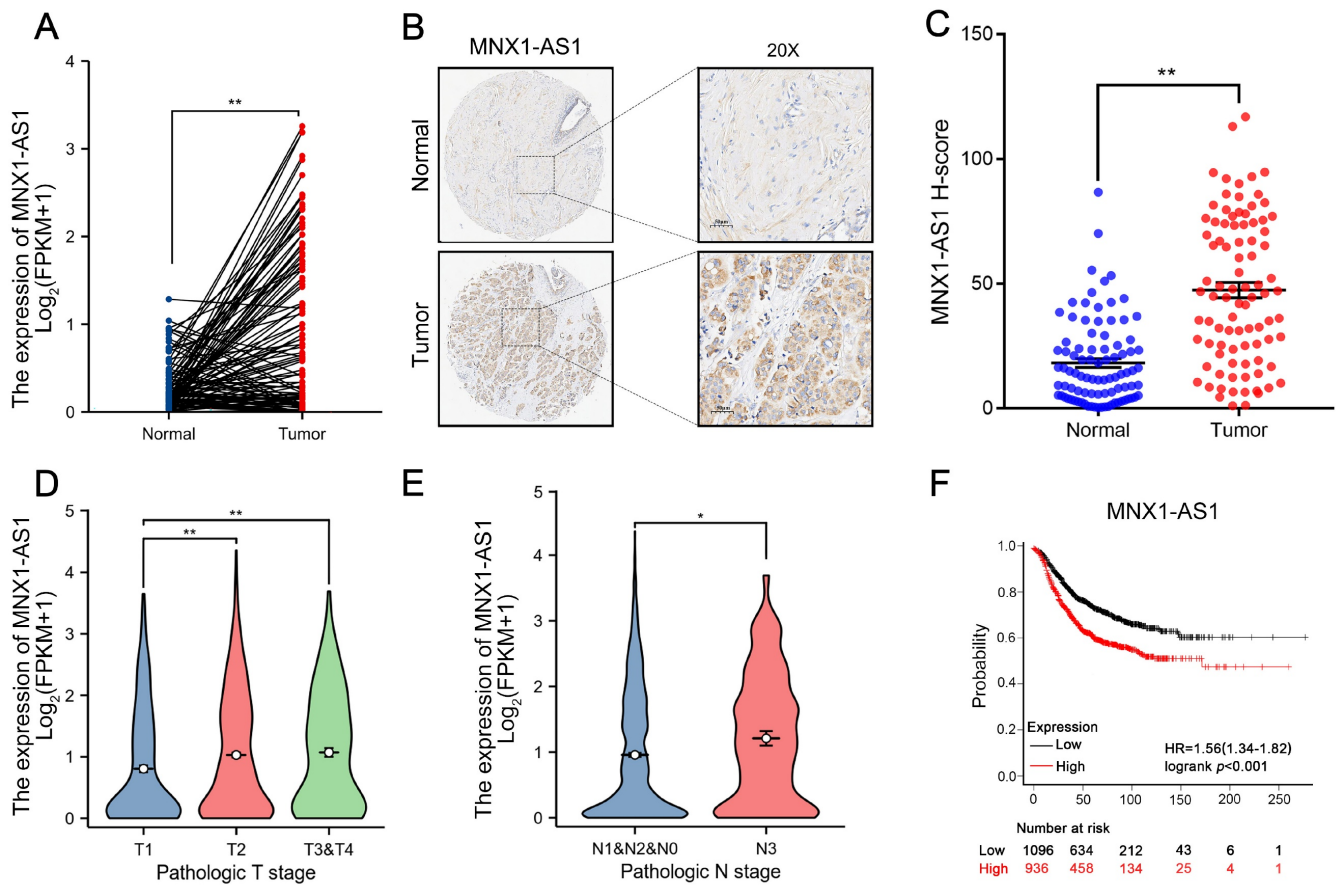
** Levels of MNX1-AS1 is significantly increased in BC tissues and associated with poor prognosis.** (A) MNX1-AS1 expression in BC tissues (n =113) and paired normal tissues (n = 113) in the TCGA dataset. (B) Representative ISH results of MNX1-AS1 expression from the BC tissue microarray. (C) Statistical analysis of ISH expression in BC tissues (n=90) and paired normal tissues (n=90). (D-E) The expression of MNX1-AS1 exhibited obvious upregulation in BC patients with a higher pathological stage according to TCGA data. (F) Kaplan-Meier analysis revealed the overall survival in BC patients based on the relative MNX1-AS1 expression. Data are presented as mean ± SEM. * p < 0.05, ** p < 0.01.

MNX1-AS1 is an antisense lncRNA located on chromosome 7 at q36.3. As shown in Figure 1A and Figure S1A, analysis of the The Cancer Genome Atlas (TCGA) data indicated a significant upregulation of MNX1-AS1 expression across multiple cancers, including BC. Notably, validation in an independent BC cohort (n=90) further confirmed that MNX1-AS1 was markedly increased in BC tissues compared with corresponding normal controls (Fig. 1B-C). Moreover, when BC patients were categorized into subtypes, including luminal A, luminal B, HER2+, and TNBC subtypes, MNX1-AS1 expression was consistently higher in all subtypes of BC compared to corresponding normal tissue samples, with no statistically significant differences observed between the subtypes (Fig. S1B). Furthermore, MNX1-AS1 overexpression was strongly associated with tumor size and lymph node metastasis in BC patients (Fig. 1D-E). Kaplan-Meier (KM) plotter analysis was employed to investigate the prognostic value of MNX1-AS1 in BC (http://kmplot.com/analysis/) Higher MNX1-AS1 expression was significantly correlated with worse overall survival (OS), underscoring the prognostic importance of MNX1-AS1 in BC (Fig. 1F). These findings indicate that MNX1-AS1 expression is markedly dysregulated in BC and closely associated with adverse clinical outcomes in patients with BC.

### MNX1-AS1 promotes the proliferation of BC cells by activating the ITGA6/PI3K/AKT pathway

**Figure 2:**
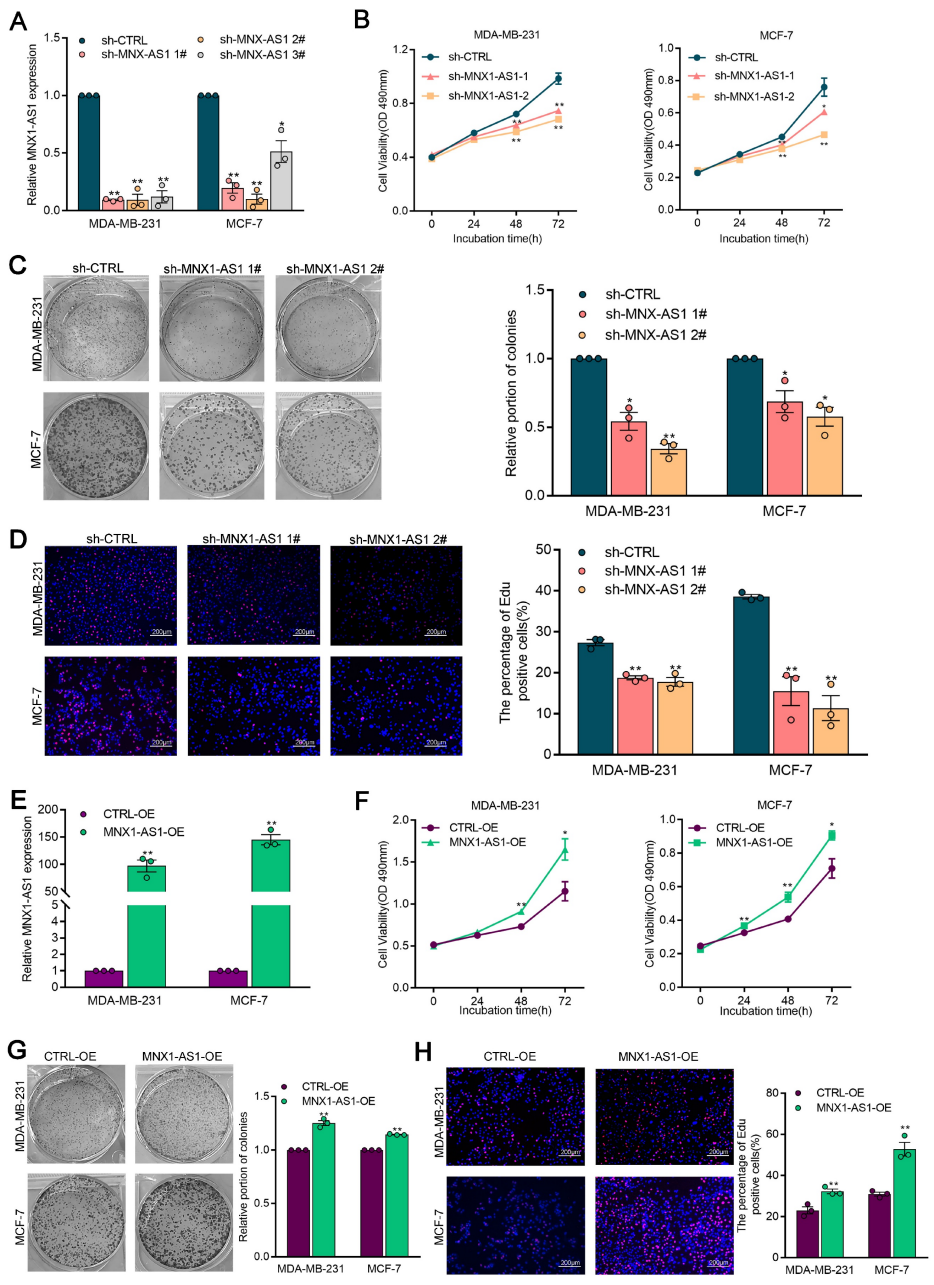
** MX1-AS1 promotes BC cell proliferation.** (A) MNX1-AS1 knockdown efficiency was analyzed by qRT-PCR assays in BC cells. (B) Cell viability examinations of BC cells with MNX1-AS1 knockdown. (C) Colony-forming assays were conducted to determine the proliferation of BC cells with MNX1-AS1 knockdown. (D) EdU staining assays were used to determine the proliferation of BC cells with MNX1-AS1 knockdown. (E) MNX1-AS1 overexpression efficiency was analyzed by qRT-PCR assays in BC cells. (F) Cell viability examinations of BC cells with MNX1-AS1 overexpression. (G) Colony-forming assays were conducted to determine the proliferation of BC cells with MNX1-AS1 overexpression. (H) EdU staining assays were used to determine the proliferation of BC cells with MNX1-AS1 overexpression. Data are presented as mean ± SEM. * p < 0.05, ** p < 0.01.

To elucidate the role of MNX1-AS1 in BC progression, we established BC cell lines with stable knockdown or overexpression of MNX1-AS1 using shRNAs or overexpression lentivirus, respectively. MTS and colony formation assays demonstrated that MNX1-AS1 knockdown significantly inhibited BC cell proliferation (Fig. 2A-2C). In addition, the proportion of EdU-positive cells (cells in the mitotic S phase), decreased following MNX1-AS1 knockdown (Fig. 2D). Conversely, stable overexpression of MNX1-AS1 induced a significant increase in cell proliferation (Fig. 2E-2H). These results indicate that MNX1-AS1 promotes BC cell proliferation and acts as an oncogene in BC.

**Figure 3:**
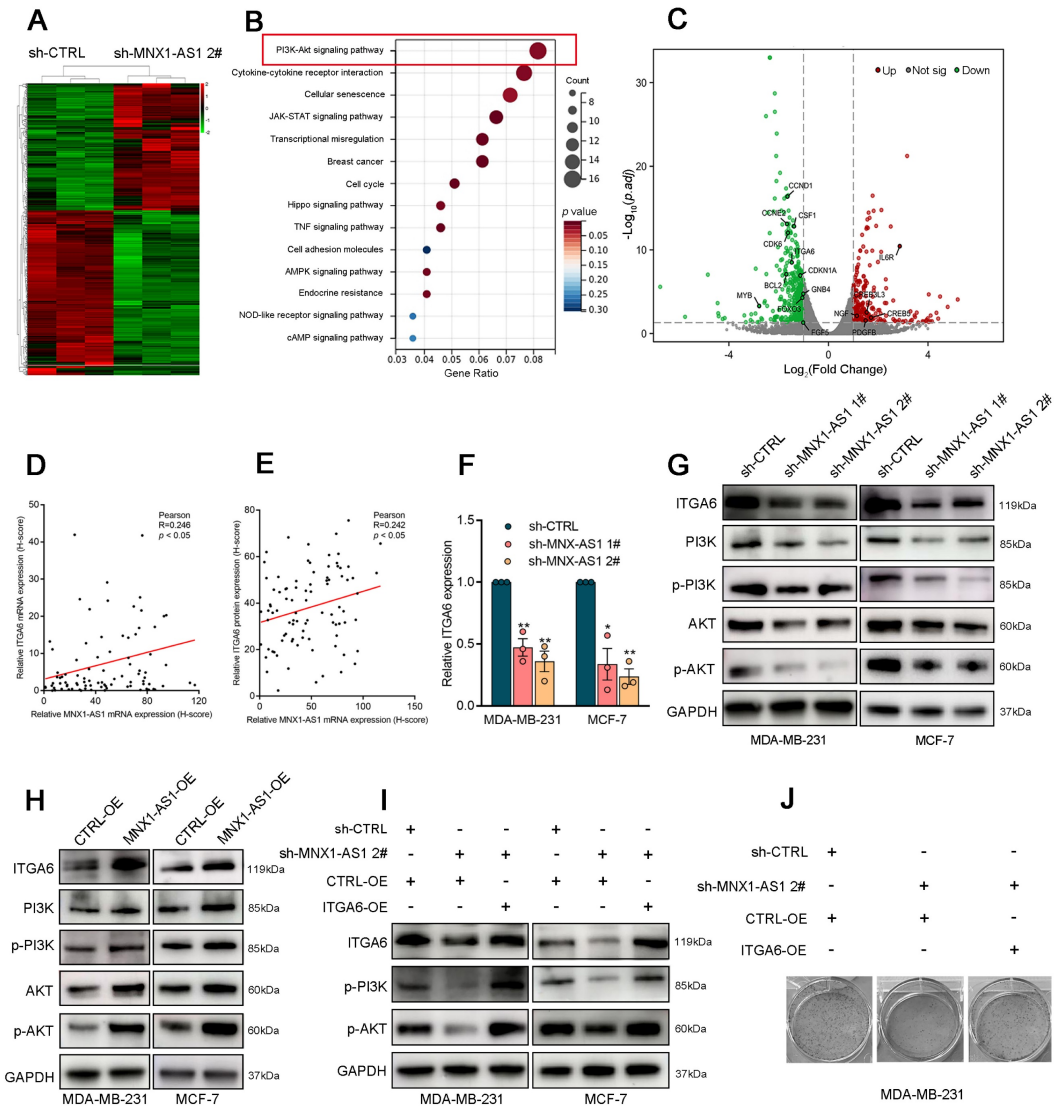
** ITGA6/PI3K/AKT is a key downstream pathway of MNX1-AS1 in BC.** (A) The heatmap of RNA transcription sequencing of the control group and the sh-MNX1-AS1 group. (B) KEGG analysis for all altered genes after knockdown of MNX1-AS1. (C) The scatter plot of RNA transcription sequencing with the genes related to PI3K/AKT pathway were labeled. (D) The correlation between ITGA6 mRNA (detected by ISH) and MNX1-AS1 mRNA expression levels (detected by ISH) in BC tissue samples. (E) The correlation between ITGA6 protein expression (detected by IHC) and MNX1-AS1 mRNA expression levels (detected by ISH) in BC tissue samples. (F) ITGA6 showed obvious decrease in BC cells with MNX1-AS1 knockdown at mRNA level. (G-H) Western blot assays confirmed that MNX1-AS1 knockdown inhibited the ITGA6/PI3K/AKT pathway, while its overexpression activated it. (I) The inhibition PI3K/AKT pathway was partially reversed by ITGA6 overexpression. (J) The inhibition of BC proliferation was partially reversed by ITGA6 overexpression. Data are presented as mean ± SEM. * p < 0.05, ** p < 0.01.

To further investigate the mechanism by which MNX1-AS1 contributes to BC progression, we performed RNA sequencing on control or MNX1-AS1-knockdown MDA-MB-231 cells (Fig. 3A). Gene Ontology (GO) analysis indicated that MNX1-AS1 is involved in various biological processes, including cell proliferation, DNA replication initiation, and cell death (Fig. S2A). Additionally, Kyoto Encyclopedia of Genes and Genomes (KEGG) enrichment analysis highlighted the critical involvement of the PI3K/AKT pathway in mediating the biological effects of MNX1-AS1 (Fig. 3B). Notably, the expression of 16 genes linked to the PI3K/AKT pathway was significantly altered in BC cells with MNX1-AS1 knockdown (Fig. 3C). Specifically, ITGA6 has emerged as a crucial activator of the PI3K/AKT pathway (Fig. S2B)^29^. Analysis of tissue samples from cohort 1 revealed significantly elevated mRNA and protein levels of ITGA6 in BC tissues as compared with adjacent normal tissues (Fig. S2C-D). Correlation analysis further revealed a positive associations between ITGA6 and MNX1-AS1 (Fig. 3D-E). As shown in Figure 3F-3G, silencing of MNX1-AS1 significantly decreased ITGA6 expression at both mRNA and protein levels in BC cells. Western blotting experiments confirmed that MNX1-AS1 knockdown inhibited the PI3K/AKT pathway, whereas MNX1-AS1 overexpression had the opposite effects (Fig. 3G-H).

Besides, rescue assays further revealed that the inhibitory effect of MNX1-AS1 knockdown on the PI3K/AKT pathway was reversed by ITGA6 overexpression (Fig. 3I). Colony formation assays demonstrated that the inhibition of BC cell proliferation induced by MNX1-AS1 knockdown was reversed by ITGA6 overexpression (Fig. 3J), suggesting that ITGA6 is essential for the suppression of BC cell proliferation induced by MNX1-AS1 knockdown. Furthermore, the AKT inhibitor ARQ-092 reversed the promoting effects of MNX1-AS1 overexpression on AKT protein expression (Fig. S2E) and BC cell proliferation (Fig. S2F). These observations highlight that MNX1-AS1 promotes BC cell proliferation by activating the ITGA6/PI3K/AKT signalling pathway.

### Silencing of MNX1-AS1 increases chemosensitivity of BC cells to paclitaxel

**Figure 4:**
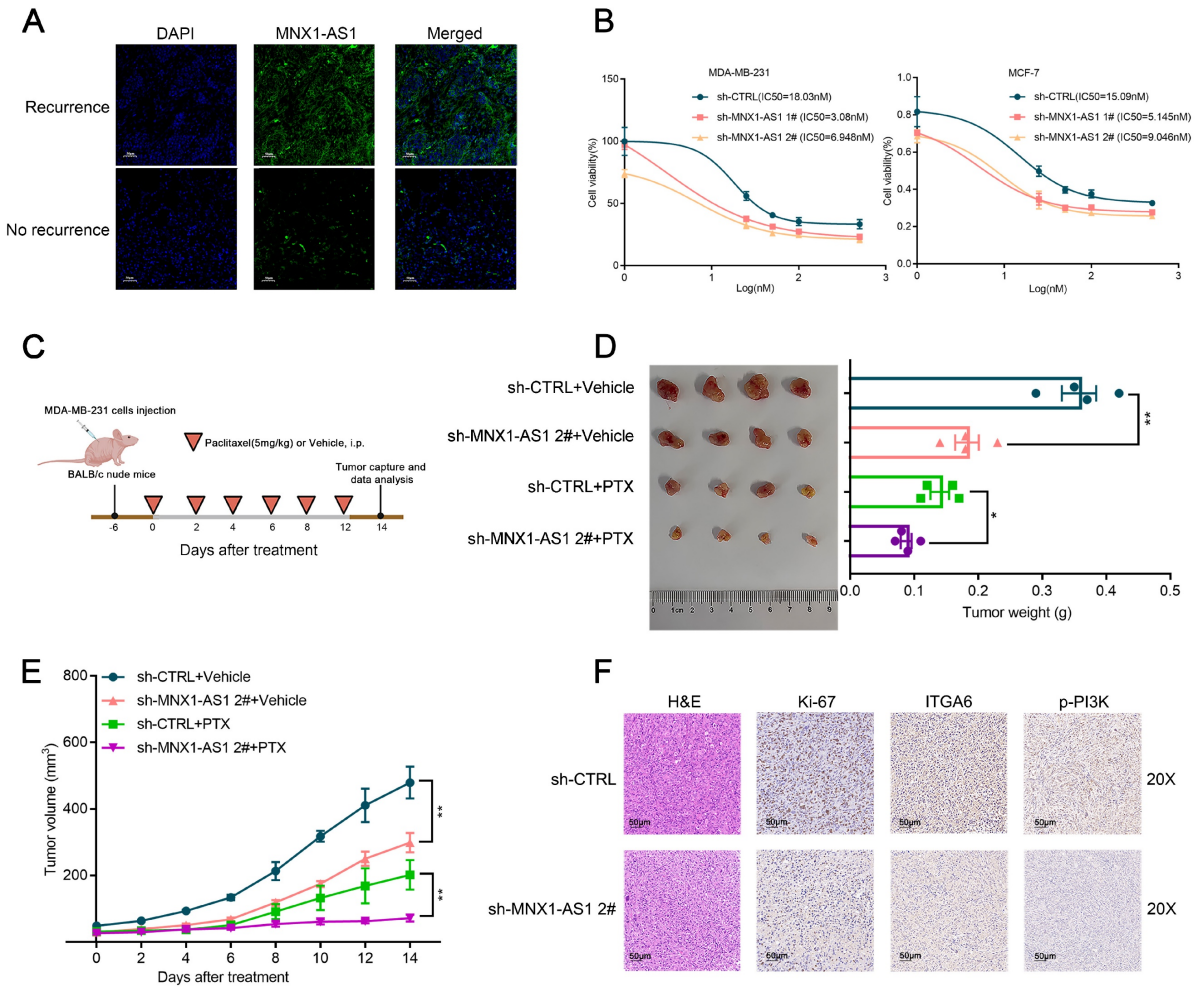
** MNX1-AS1 accelerates BC proliferation and enhances the resistance of BC cells to paclitaxel.** (A) Representative FISH results of MNX1-AS1 expression from the BC tissue with or without recurrence. (B) Changes in IC50 values of the paclitaxel in BC cells after MNX1-AS1 knockdown. (C-E) MDA-MB-231 cells stably expressing sh-CTRL and sh-MNX1-AS1 were inoculated into BALB/c female nude mice. After 6 days of injection, DMSO or paclitaxel (5 mg/kg, dissolved in DMSO) was administered by intraperitoneal injection every 2 days. Representative tumor images (C), tumor weight (D), and tumor volume (E) were shown. (F) Tumor tissue samples were immunostained for H&E, Ki-67, ITGA6 and p-PI3K. Data are presented as mean ± SEM. * p < 0.05, ** p < 0.01.

While several novel therapeutic options have emerged for BC, chemotherapy remains a cornerstone of treatment^30^,^31^. Recent advances have highlighted the role of the PI3K/AKT pathway in modulating the sensitivity of tumor cells to chemotherapy treatments^27^,^28^. However, the role of MNX1-AS1 in regulating drug sensitivity remains unexplored. To further elucidate the function of MNX1-AS1 in chemotherapy resistance in BC, we performed FISH assays to analyse clinical samples. Among 17 BC patients who received adjuvant chemotherapy after surgery, those with postoperative recurrence exhibited relatively higher MNX1-AS1 expression (Fig. 4A, Fig. S3A). Moreover, the analysis of sequencing expression profiles of tissues from chemotherapy-sensitive and resistant BC patients revealed that MNX1-AS1 was markedly upregulated in the resistant samples (GSE221060, Fig. S3B). These results provide the first evidence that MNX1-AS1 plays a pivotal role in regulating the chemosensitivity in BC cells. The key chemotherapy agents used in BC chemotherapy include paclitaxel, doxorubicin (DOX), and 5-fluorouracil (5-FU)^32^. To further evaluate whether MNX1-AS1 affects BC cell sensitivity to paclitaxel, we conducted both in vitro and in vivo experiments. As depicted in Figure 4B, the half-maximal inhibitory concentration (IC50) values of paclitaxel were obviously lower in the MNX1-AS1 knockdown group compared to the control group. Silencing of MNX1-AS1 markedly decreased the IC50 values of 5-FU and DOX in BC cells (Fig. S3C).

In vivo experiments demonstrated that MNX1-AS1 knockdown not only significantly suppressed tumor growth but also enhanced the cytotoxicity of paclitaxel in BC cell xenografts. Specifically, the reduction in tumor volume was more pronounced in the MNX1-AS1 knockdown group compared to the control group. Moreover, the combination of MNX1-AS1 knockdown and paclitaxel treatment led to greater inhibition of tumor growth than either treatment alone (Fig. 4C-E). These findings suggest that targeting MNX1-AS1 could potentiate the efficacy of paclitaxel in BC therapy.

Moreover, Ki-67 staining of tumor slices indicated that cell proliferation was significantly inhibited in the MNX1-AS1-knockdown group (Fig. 4F). Consistent with these observations, MNX1-AS1 knockdown led to a marked decrease in the expression of ITGA6 and p-PI3K in MDA-MB-231 xenografts (Fig. 4F). These results indicate that MNX1-AS1 knockdown increases the sensitivity of BC cells to paclitaxel by blocking the PI3K/AKT pathway. Consistent with these findings, MNX1-AS1 knockdown led to a marked decrease in the expression of ITGA6 and p-PI3K in MDA-MB-231 xenografts (Fig. 4F). These results suggest that MNX1-AS1 knockdown increases the sensitivity of BC cells to paclitaxel by blocking the PI3K/AKT pathway.

### MNX1-AS1 binds to YBX1 and increases its expression by inhibiting its ubiquitination

**Figure 5:**
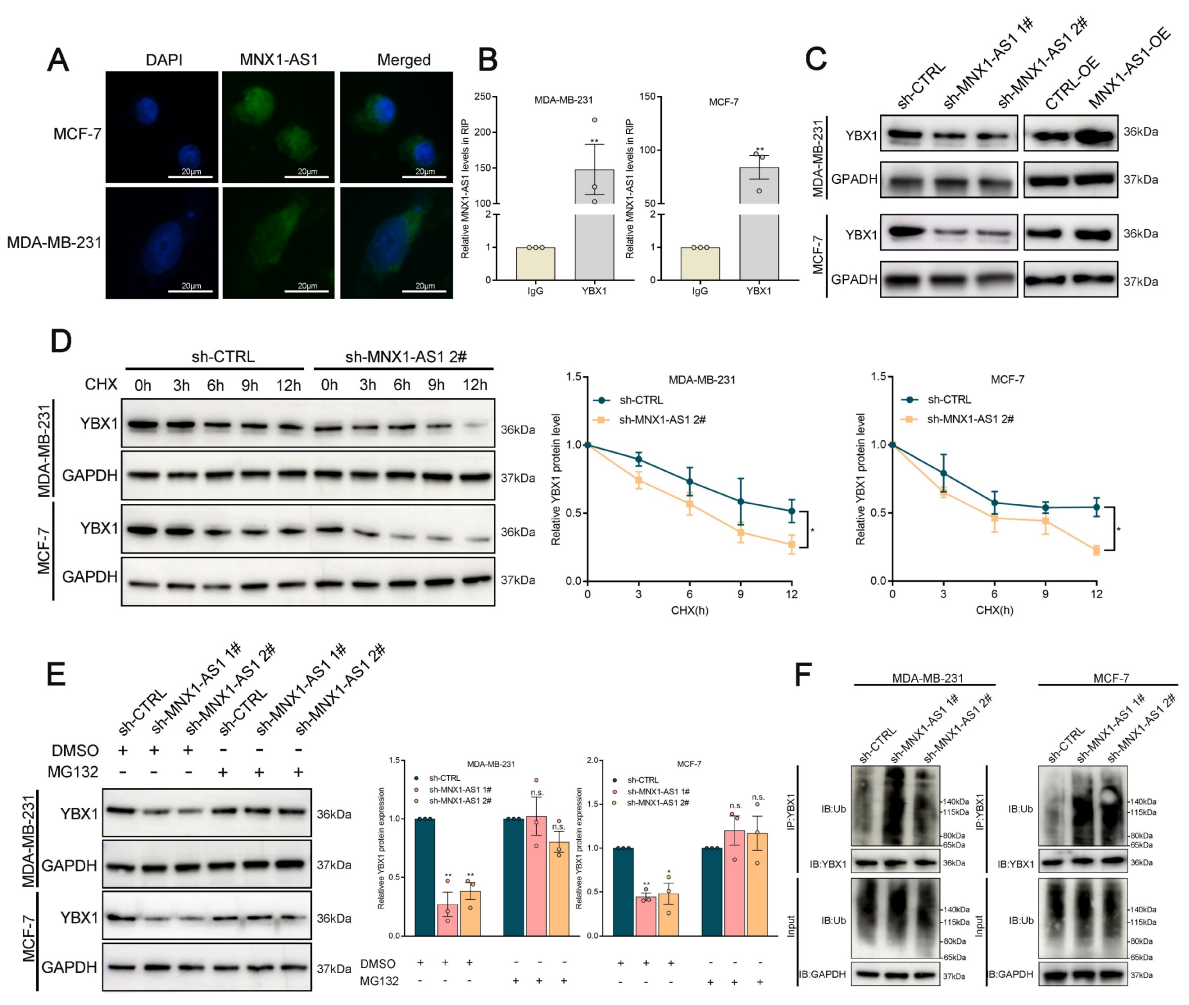
** MNX1-AS1 binds with YBX1 and decreases the ubiquitination of YBX1.** (A) FISH assays were used to determine the distribution of MNX1-AS1 in MDA-MB-231 and MCF-7 cells. (B) The RIP assays revealed the enrichment of MNX1-AS1 in YBX1 RIP, as compared with its matched IgG group. (C) Western blot analysis of the expression of YBX1 in BC cells with MNX1-AS1 knockdown and overexpression. (D) Relative YBX1 protein levels in MNX1-AS1-silenced cells following CHX treatment. (E) Relative YBX1 protein levels in MNX1-AS1-silenced cells with or without MG132 treatment. (F) Immunoblotting for ubiquitin following immunoprecipitation with YBX1 antibody in MNX1-AS1 knockdown or control cells treated with MG132. Data are presented as mean ± SEM. * p < 0.05, ** p < 0.01.

To investigate the molecular mechanism by which MNX1-AS1 regulates BC progression, we determined its subcellular distribution of MNX1-AS1 in BC cells through FISH and subcellular fractionation localization assays (Fig. 5A, Fig. S4A). We observed that MNX1-AS1 was distributed in both the cytoplasm and nucleus, suggesting its regulatory effects at both transcriptional and posttranscriptional levels. Recent studies have shown that lncRNAs can regulate tumor phenotypes through interactions with RNA-binding proteins (RBPs). YBX1, also referred to as YB-1, is essential for the progression and drug resistance of various cancers and interacts with numerous noncoding RNAs to influence their biological activities^33^-^35^. According to the catRAPID database, the interaction propensity between MNX1-AS1 and YBX1 is 26.93, revealing the close association between MNX1-AS1 and YBX1. Subsequently, RNA immunoprecipitation (RIP) experiments confirmed that MNX1-AS1 binds to YBX1 in BC cells (Fig. 5B).

Furthermore, MNX1-AS1 silencing significantly attenuated YBX1 protein expression without altering its mRNA levels (Fig. 5C, Fig. S4B). MNX1-AS1-depleted cells exhibited accelerated YBX1 protein degradation compared with controls under the protein synthesis inhibitor cycloheximide (CHX) (Fig. 5D). These results indicate that MNX1-AS1 may regulate the stability of YBX1 protein. To further investigate whether MNX1-AS1 regulates the stability of YBX1 protein through a proteasome-dependent pathway, the proteasome inhibitor MG132 was implicated in this study. MG132 treatment markedly rescued YBX1 protein levels in MNX1-AS1-depleted cells to control levels (Fig. 5E). The ubiquitin‒proteasome system (UPS) enhances the proteasomal degradation of its target proteins. Therefore, we investigated whether MNX1-AS1 regulates the degradation of YBX1 protein via the UPS. As shown in Fig. 5F, ubiquitinated YBX1 levels were markedly elevated in MNX1-AS1-depleted cells as compared with controls (Fig. 5F). These observations suggest that MNX1-AS1 inhibits the ubiquitination of YBX1 and protects it from proteasomal degradation, thereby increasing YBX1 protein stability in BC cells.

### MNX1-AS1 stabilizes YBX1 by disrupting its interaction with SMURF2

**Figure 6:**
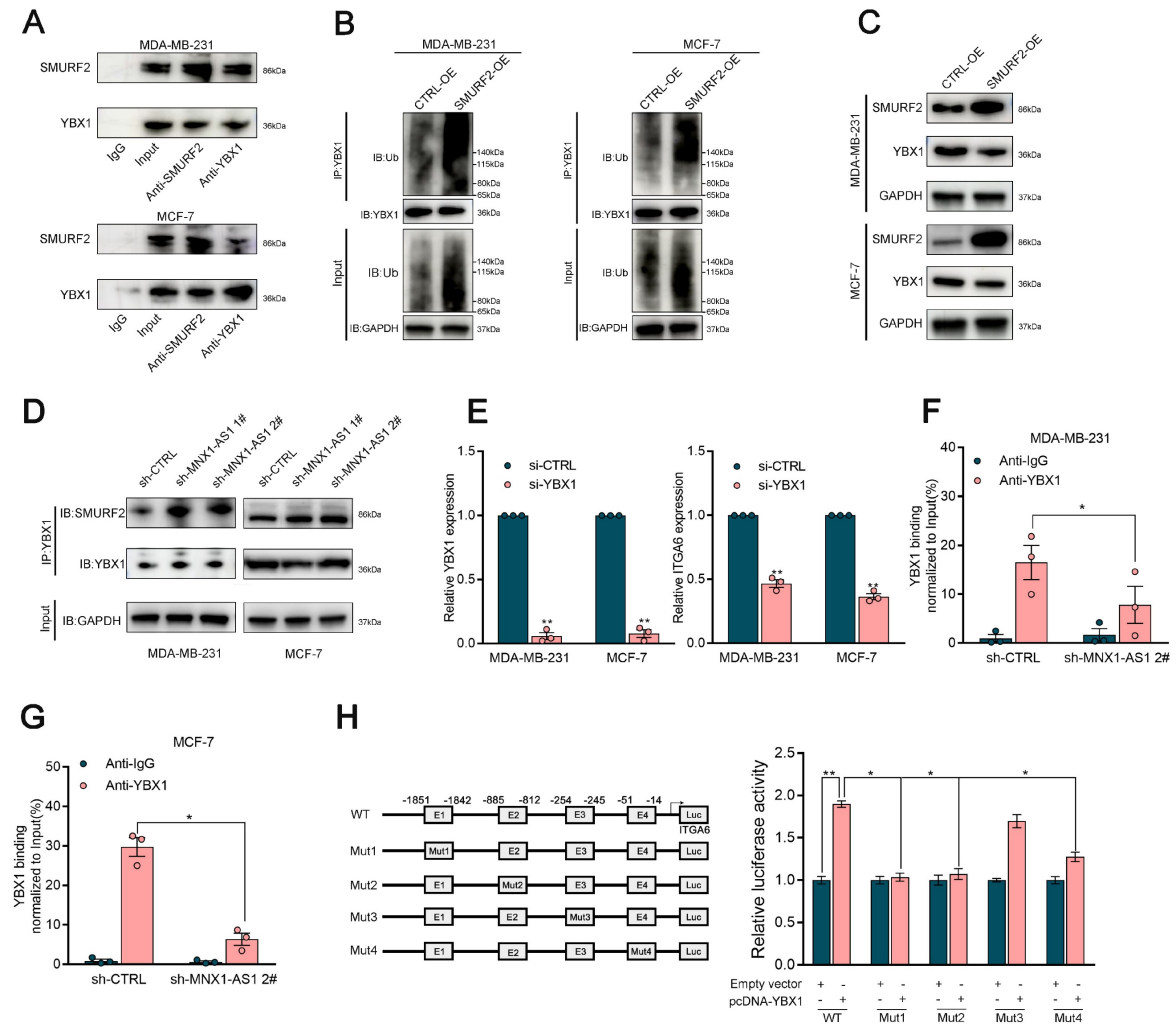
** MNX1-AS1 disrupts the binding of SMURF2 with YBX1.** (A) Immunoblotting for YBX1 and SMURF2 following immunoprecipitation with SMURF2 and YBX1 antibody. (B) Immunoblotting for ubiquitin following immunoprecipitation with YBX1 antibody in cells stably overexpressing SMURF2 and controls, treated with MG132. (C) Relative levels of YBX1 protein in SMURF2 overexpressing cells or control cells. (D) Western blot analysis of SMURF2 and YBX1 in Co-IP assays performed with control and MNX1-AS1-silenced BC cells. (E) The qRT-PCR analysis of ITGA6 expression in BC cells with YBX1 knockdown. (F-G) ChIP assays demonstrated that knockdown of MNX1-AS1 reduces YBX1 enrichment in the promoter region of ITGA6. (H) Dual-luciferase reporter assays revealed that YBX1 binds to the E1, E2 and E4 promoter region of ITGA6. Error bars show the SD from three independent experiments. Data are presented as mean ± SEM. * p < 0.05, ** p < 0.01.

We further explored the potential ubiquitinating enzymes that might influence the stability of the YBX1 protein. According to the HitPredict database, a potential interaction between YBX1 and Smad-specific E3 ubiquitin ligase 2 (SMURF2) was identified. (Interaction score=0.479). To validate this hypothesis, we performed coimmunoprecipitation (Co-IP) assays, which demonstrated a close association between SMURF2 and YBX1, with significantly higher YBX1 enrichment in SMURF2 antibody relative to IgG controls (Fig. 6A). Next, further investigation was conducted to determine whether SMURF2 influences the ubiquitination of YBX1. It was elucidated that ectopic expression of SMURF2 substantially increased the ubiquitination of YBX1, revealing the critical involvement of SMURF2 in mediating the ubiquitination of YBX1 (Fig. 6B).

Specifically, ectopic SMURF2 expression elicited a marked decrease in YBX1 protein abundance, indicative of SMURF2-mediated degradation of YBX1 through ubiquitin‒proteasome pathway (Fig. 6C). It is notable that MNX1-AS1 did not affect SMURF2 protein expression (Fig. S4C). However, co-IP assays revealed that silencing MNX1-AS1 in BC cells enhanced the interaction between YBX1 and SMURF2 (Fig. 6D). These findings indicate that MNX1-AS1 stabilizes YBX1 by disrupting its interaction with SMURF2, thereby preventing its ubiquitin-dependent degradation.

### MNX1-AS1 critically activates ITGA6 expression at the transcriptional level by enhancing the binding of YBX1 to its promoter

As shown previously, MNX1-AS1 stabilizes YBX1 by preventing its interaction with SMURF2. Overexpression of MNX1-AS1 significantly increased ITGA6 levels. It is known that YBX1 can function as a transcription factor by directly binding to the promoter regions of target genes, enhancing the recruitment of the transcriptional machinery and increasing gene expression^36^. Based on these findings, we hypothesized that YBX1 may be involved in the activation of ITGA6 induced by MNX1-AS1. To verify this hypothesis, we initially determined the regulatory effects of YBX1 on ITGA6 expression, illuminating that YBX1 depletion significantly decreased ITGA6 expression in BC cells (Fig. 6E). Bioinformatics analysis using JASPAR identified multiple putative binding sites for YBX1 within the ITGA6 promoter region. ChIP‒qPCR assays demonstrated that MNX1-AS1 silencing led to a significant decrease in YBX1 occupancy in the ITGA6 promoter region (Fig. 6F‒G). Furthermore, dual-luciferase reporter assays revealed that YBX1 overexpression markedly increased the luciferase activities of the Luc-WT and Luc-Mut3 reporters, while showing no significant effects on Luc-M1, Luc-Mut2, or Luc-Mut4 reporters (Fig. 6H). Taken together, these findings indicate that depletion of MNX1-AS1 impairs YBX1 binding to the ITGA6 promoter region and reduces the transcriptional activation of ITGA6 in BC cells.

### MNX1-AS1 functions to stabilize ITGA6 mRNA in an m6A-dependent manner through its cooperation with IGF2BP2

**Figure 7:**
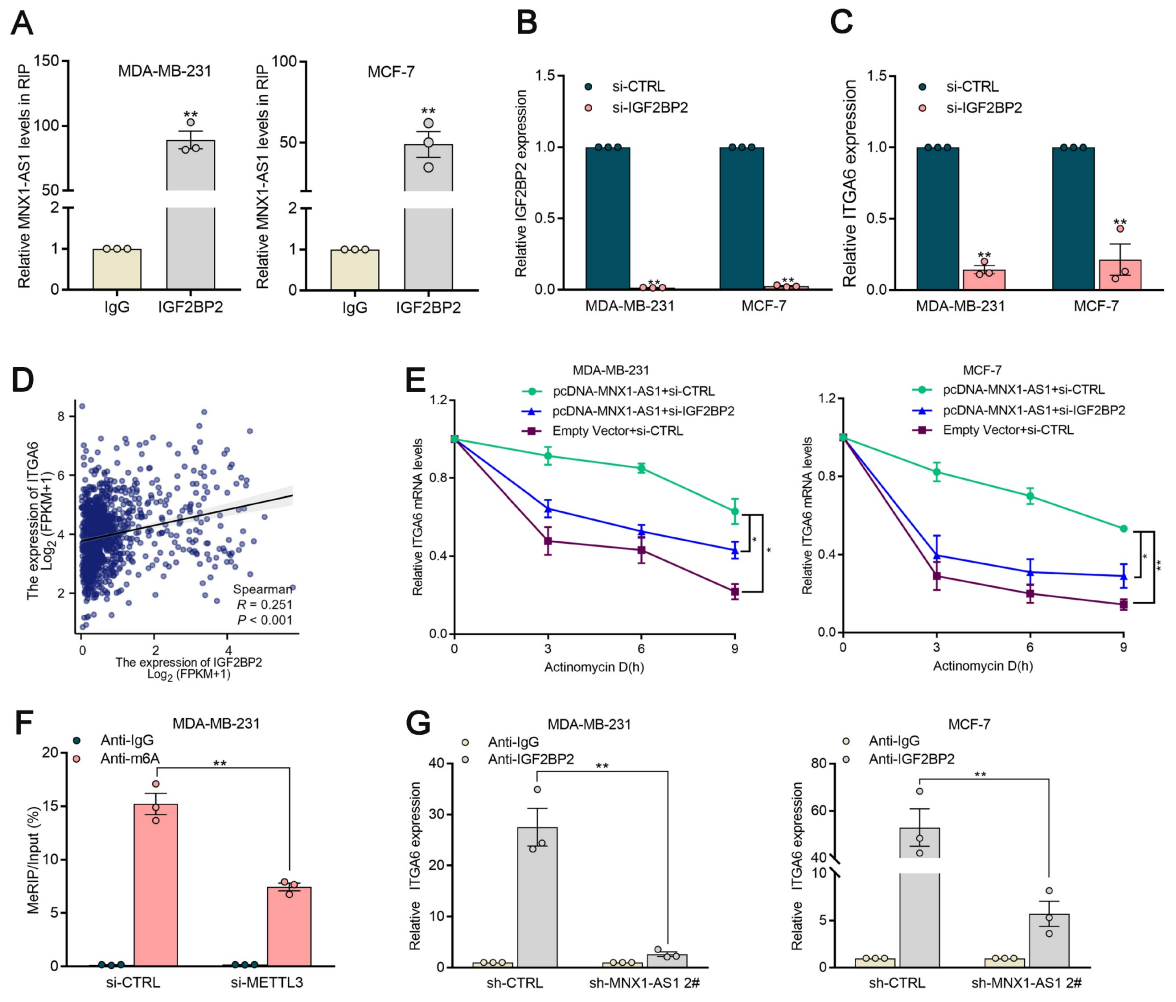
** MNX1-AS1 cooperates with IGF2BP2 to regulate ITGA6 expression in an m6A-dependent manner.** (A) The RIP assays revealed the enrichment of MNX1-AS1 in IGF2BP2 RIP, as compared with its matched IgG group. (B-C) Relative IGF2BP2 and ITGA6 mRNA levels in cells with IGF2BP2 knockdown. (D) The correlation between IGF2BP2 and ITGA6 was assessed using TCGA data analysis. (E) RNA stability assays were performed using Actinomycin D (2μg/ml) to disrupt RNA synthesis in BC cells, and the degradation rates of the ITGA6 mRNAs were measured every 3 h by qRT-PCR. (F) MeRIP-qPCR assays were performed to quantify the relative m6A modification level of ITGA6 upon MNX1-AS1 knockdown in BC cells. (G) The RIP assays was performed in MNX1-AS1-silenced and control BC cells. The coprecipitated RNA was analyzed by qRT-PCR for ITGA6. The fold enrichment of ITGA6 in IGF2BP2 RIP was compared to the IgG group. Data are presented as mean ± SEM. * p < 0.05, ** p < 0.01.

Recent studies have revealed that m6A modification plays a crucial role in regulating the stability of mRNA transcripts^37^. Bioinformatics approaches were used to predict proteins that may interact with MNX1-AS1. MNX1-AS1 was predicted to strongly interact with IGF2BP2, an m6A methylation reader (Table S1). Thus, RIP assays were conducted to further investigate the interaction between MNX1-AS1 and IGF2BP2 in BC cells (Fig. 7A). It was elucidated that MNX1-AS1 could bind with IGF2BP2 in BC cells. In addition, the alteration of MNX1-AS1 exhibited no significant effects on IGF2BP2 protein abundance in BC cells (Fig. S4C).

Compelling studies have highlighted that IGF2BP2 serves as an m6A reader, mediating the stabilization of various mRNA transcripts^38^. Building upon the elucidated regulatory axis wherein ITGA6 functions as a direct downstream effector of MNX1-AS1, we further determine whether MNX1-AS1 works in tandem with IGF2BP2 to mediate the stability of ITGA6. It was observed that the depletion of IGF2BP2 induced a significant reduction in ITGA6 mRNA levels (Fig. 7B‒C). Meanwhile, integrative analysis of TCGA database further confirmed a positive correlation between IGF2BP2 and ITGA6 in BC tissue samples (Fig. 7D).

To clarify the potential role of MNX1-AS1/IGF2BP2 in stabilizing ITGA6, we used actinomycin D and found that the absence of IGF2BP2 eliminated the steady increase in ITGA6 expression induced by MNX1-AS1 overexpression (Fig. 7E). Next, we further determine whether ITGA6 undergoes m6A methylation, MeRIP‒qPCR assays were conducted to determine whether the 3' untranslated region (UTR) of the ITGA6 transcript was reduced in METTL3-depleted BC cells (Fig. 7F). It was detected that MNX1-AS1 silencing did not significantly alter the expression of METTL3, METTL4, or WTAP in BC cells. These observations illuminated that MNX1-AS1 does not regulate ITGA6 expression through direct modulation of m6A writer machinery. Furthermore, RIP‒qPCR assays revealed that MNX1-AS1 knockdown significantly attenuated the interaction between IGF2BP2 and ITGA6, highlighting that MNX1-AS1 cooperates with IGF2BP2 to increase the stability of ITGA6 in BC cells (Fig. 7G).

### LNP-si-MNX1-AS1 exert a strong antitumor effect on BC cells in vivo

**Figure 8:**
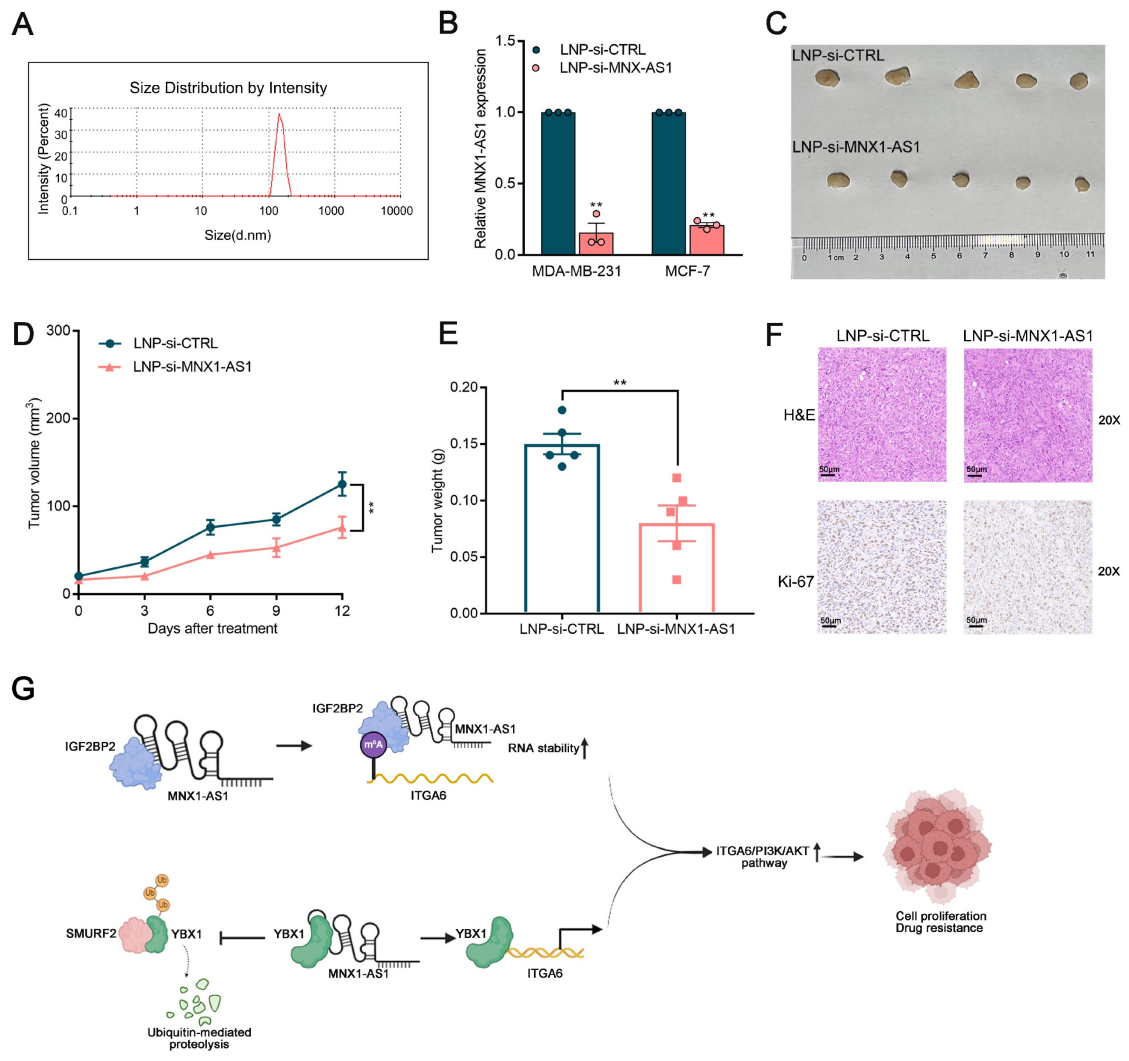
** Therapeutic efficacy and toxicity evaluation of systemic injection of LNP (si-MNX1-AS1).** (A) The size distribution profile of LNPs (si-MNX1-AS1). (B) The qRT-qPCR assays were performed to evaluate the expression level of MNX1-AS1 between LNP-si-MNX1-AS1 and LNP-si-CTRL. (C-E) MDA-MB-231 cells were inoculated into BALB/c female nude mice. Three days after injection, LNP-si-CTRL or LNC-si-MNX1-AS1 was injected peritumorally every three days. Representative tumor images (C), tumor volume (D), and tumor weight (E) are shown. (F) Tumor tissue samples were immunostained with H&E and Ki-67. (G) The schematic model of the contributor and regulatory mechanism of MNX1-AS1 in the occurrence and chemotherapy resistance of BC. (Figure was created with BioRender.com). Data are presented as mean ± SEM. * p < 0.05, ** p < 0.01.

To explore the potential therapeutic utility of MNX1-AS1 in BC, lipid nanoparticles (LNPs) loaded with si-MNX1-AS1 and si-control were generated via a hybrid microfluidics method. Specifically, the mean diameter of LNP-si-MNX1-AS1 was 356.6 nm, with a polydispersity value of 0.389 (Fig. 8A). As shown in Figure 8B, the expression of MNX1-AS1 was significantly reduced in BC cells treated with LNP-si-MNX1-AS1. We subcutaneously injected BC cells into nude mice to establish xenograft tumor models and treated the mice with LNP-si-MNX1-AS1 or LNP-si-CTRL. The results revealed that LNP-si-MNX1-AS1 treatment significantly suppressed tumor growth as compared with the controls. Furthermore, tumor volume and weight were significantly inhibited in the LNP-si-MNX1-AS1 treatment group (Fig. 8C-8E). The results of Ki-67 staining of tumor slices indicated that the proliferative capacity was significantly lower in the MNX1-AS1-knockdown group realtive to the control group (Fig. 8F). Additionally, we assessed systemic toxicity through H&E staining and revealed that intravenous delivery of LNP-si-MNX1-AS1 had no significant effect on major organs, such as the liver, kidneys, lungs, spleen, or heart (Fig. S5A). The safety of LNP-si-MNX1-AS1 was validated. These results indicate that the nanoparticle delivery system provides an efficient and safe method for delivering MNX1-AS1 siRNA, offering a mechanistic foundation for clinical translation of RNAi-based precision therapy for BC patients.

## Discussion

MNX1-AS1 has been identified as a key regulatory factor in the pathogenesis of several malignant tumors. However, the mechanisms by which MNX1-AS1 affects BC progression remain largely unknown. Importantly, the role of MNX1-AS1 in regulating BC chemosensitivity and the effectiveness of targeting MNX1-AS1 as a therapeutic approach in BC have yet to be elucidated. The present study revealed that MNX1-AS1 was markedly dysregulated in BC tissue samples and significantly correlated with postoperative recurrence rates. Both in-vitro and in-vivo validation experiments revealed that silencing of MNX1-AS1 obviously inhibited BC cell growth and enhanced the chemosensitivity of BC cells. To further elucidate the underlying regulatory mechanism of MNX1-AS1 in BC, we conducted RNA-seq assays and revealed that MNX1-AS1 was involved in activating the ITGA6/PI3K/AKT pathway. The localization of MNX1-AS1 in both the nucleus and cytoplasm highlighted its role in promoting BC carcinogenesis through mechanisms at the transcriptional and post-transcriptional levels.

Mechanistic studies revealed that MNX1-AS1 can bind to YBX1 and competitively prevent its interaction with SMURF2, thus blocking the degradation of YBX1 and activating its expression. SMURF2, an E3 ubiquitin ligase belonging to the HECT family, plays a crucial role in regulating protein degradation via the ubiquitin-proteasome system and participates in multiple cellular functions, such as transcription regulation and intracellular transport^39^. This study not only identified YBX1 as a novel target protein of SMURF2 but also elucidated a novel mechanistic model of the MNX1-AS1/SMURF2/YBX1 axis. We further demonstrated that YBX1 can act as a transcription factor by directly binding to the promoter of ITGA6, increasing ITGA6 expression and subsequently activating the PI3K/AKT pathway. In addition, lncRNA-mediated posttranscriptional regulation plays a crucial role in gene expression dynamics. It serves as a key mechanism that significantly impacts mRNA stability, translation efficiency, and the adaptation of cellular responses across diverse physiological contexts. m6A, the predominant internal modification in eukaryotic mRNAs, is known to participate in a wide range of physiological processes and performs multiple cellular functions^40^-^42^. Among the m6A regulatory factors, IGF2BP2 acts as an m6A reader that targets eukaryotic RNA to regulate its stability. Although current evidence indicates that IGF2BP2 may be involved in the regulation of target gene stability induced by lncRNA, it is still unclear whether IGF2BP2 participates in the regulation of ITGA6 mediated by MNX1-AS1^43^. Specifically, we demonstrated that MNX1-AS1 can bind to IGF2BP2 and cooperatively stabilize ITGA6 in a m6A-dependent manner, consequently triggering the activation of the PI3K/AKT pathway. These findings greatly enrich the understanding of the involvement of MNX1-AS1 in modulating epigenetic modifications, thereby improving our understanding of the molecular mechanisms underlying BC carcinogenesis and revealing a new strategy for targeted clinical intervention.

Notably, compared with conventional therapies such as small molecules and antibodies, oligonucleotide strategies offer unique advantages for targeting specific RNAs. RNAi has been identified as the most straightforward approach for silencing genes closely associated with malignant diseases. Nanotechnology-based systems represent the most effective approach for directly delivering siRNA therapies to tumor cells. We elucidated that this advanced nanotechnology-based system can be used to effectively load MNX1-AS1 siRNA into LNPs and that these LNPs significantly inhibit tumor growth in vivo, offering a new strategy for BC treatment. This study has certain limitations. For example, it is unclear whether MNX1-AS1 exerts its effects through interactions with other proteins and whether ITGA6 regulates additional downstream signalling pathways involved in tumor progression. These aspects warrant further investigation in future studies.

Overall, we highlighted MNX1-AS1 as a novel regulator closely associated with BC development and chemotherapy resistance. Moreover, the molecular mechanisms underlying BC development were elucidated. Specifically, we found that coactivation of the PI3K/AKT pathway is driven by the MNX1-AS1/SMURF2/YBX1/ITGA6 and MNX1-AS1/IGF2BP2/ITGA6 signalling axes. The developed RNAi nanoparticle system enables efficient delivery of MNX1-AS1 siRNA, underscoring the critical therapeutic role of MNX1-AS1 in BC and establishing a novel strategy for BC therapeutics (Fig. 8G).

## Conclusion

Overall, we identified MNX1-AS1 as a promising biomarker implicated in BC cell proliferation and treatment response. MNX1-AS1 enhances resistance to various chemotherapeutic agents and contributes to the tumorigenesis of BC. Interestingly, we revealed a novel mechanism in which MNX1-AS1 induces ITGA6 expression by binding to YBX1, preventing its degradation by the E3 ubiquitin ligase SMURF2. This inhibition promotes YBX1-mediated transcriptional activation of ITGA6, subsequently activating the PI3K/AKT pathway in BC. Moreover, MNX1-AS1 can interact with IGF2BP2 to increase ITGA6 stability at the posttranslational level. In summary, our study identifies MNX1-AS1 as a key mediator of chemotherapy resistance in BC and delineates its underlying molecular mechanisms. These findings establish MNX1-AS1 as a novel therapeutic target for BC and support its potential application in nanotherapy for BC.

## Supplementary Material

Supplementary experimental section, figures and tables.

## Abbreviations

BC: Breast cancer
PI3K: Phosphoinositide 3-kinase
LncRNAs: Long noncoding RNAs
m6A: N6-methyladenosine
LNPs: Lipid nanoparticles
CHX: Cycloheximide
FISH: Fluorescence in situ hybridization
YBX1: Y-box binding protein 1
SMURF2: Smad specific E3 ubiquitin ligase 2
RIP: RNA immunoprecipitation
CDX: Cell-derived xenograft
RNAi: RNA interference
