# Supplementary material for: MNX1-AS1 suppresses chemosensitivity by activating the PI3K/AKT pathway in breast cancer
Source: Int J Biol Sci. 2025 May 27;21(8):3689–704. doi: 10.7150/ijbs.104483 (PMC12160916; doi:10.7150/ijbs.104483)
Supplement: Supplementary file 1 — Supplementary experimental section, figures and tables. [file ijbsv21p3689s1.pdf]

Supplementary Materials for

MNX1-AS1 suppresses chemosensitivity by activating the PI3K/AKT pathway in breast cancer

You Shuai[1#], Zhonghua Ma[2#], Jian Yue[3#], Chunxiao Li[1], Jie Ju[1], Xue Wang[3], Haili Qian[4*], Peng Yuan[3*]

#These authors contributed equally to this work.

*Corresponding authors:

Peng Yuan, Email:yuanpeng01@cicams.ac.cn, yuanpengyp01@163.com.

Haili Qian, Email: qianhaili001@163.com.

This file includes: Supplemental Experimental Section

Supplemental Figure 1 to 5

Supplemental Table 1 to 2

Supplemental Experimental Section

Cell transfection

Stable knockdown of MNX1-AS1 was achieved in MDA-MB-231 and MCF-7 cells by lentivirus-mediated RNA interference using validated shRNA sequences inserted in the lentivector PGMLV-SC5. Randomized oligonucleotides were subcloned in the same vector and used as a control. Human DNA fragment encoding MNX1-AS1 was synthesized and incorporated into pCHD and pcDNA3.1 vectors to generate stable and transient overexpression plasmids, respectively, with the corresponding empty vectors used as controls. Small interfering RNA (siRNA) and corresponding negative control siRNA (si-CTRL) purchased from RiboBio (Beijing, China). Details are available in Table S2.

As regards transient transfection, target cells were seeded in a 6-well plate and incubated overnight at 37 °C. According to the manufacturer's instructions, use Lipofectamine 3000 (ThermoFisher, Waltham, MA, USA) for transfection.

As regards recombinant lentiviral particle production and infection, HEK-293T cells were co-transfected with knockdown or overexpression constructs and packaging plasmids using Lipofectamine 3000 as described above. Virus-containing supernatants were collected after transfection, filtered, and supplemented with 5 μg/ml polybrene to infect target cells at 48 h and 72 h. After 24 h from the second infection, target cells were selected using 1.5 μg/mL puromycin dissolved in the medium for at least 7 days.

EdU

Cells were seeded in 96-well plates at a density of $5\times10^{\wedge 3}$ cells per well. Forty-eight hours post-transfection, cells were incubated with 50 μM EdU for 2 hours at 37°C in a 5% CO_2 atmosphere. An EdU labeling/detection kit (Ribobio, Guangzhou, China) was used according to the protocol

of manufacturer.

RNA stability assays

The cells were treated with 10 μg/mL actinomycin D (MedChemExpress, USA), then collected at 0, 3, 6 and 9 hours after treatment. Total RNA was extracted and detected by qRT-PCR. Each assay was replicated three times.

Supplemental figures and figure legends

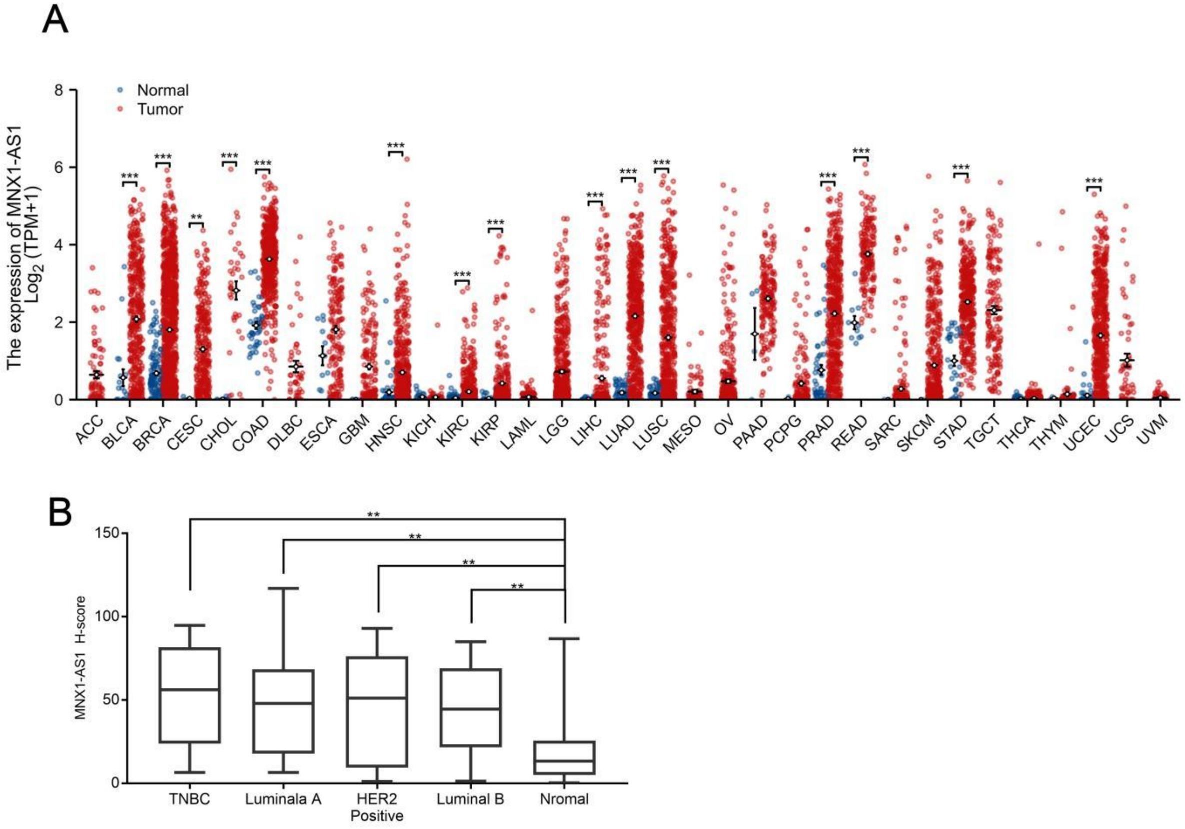

Figure. S1.

(A) Analysis of MNX1-AS1 in tumor tissues compared with normal tissues was analyzed using TCGA data.

(B) Statistical analysis of ISH expression in BC subtypes and normal tissues.

Data are presented as mean ± SEM. *p < 0.05, ** p < 0.01.

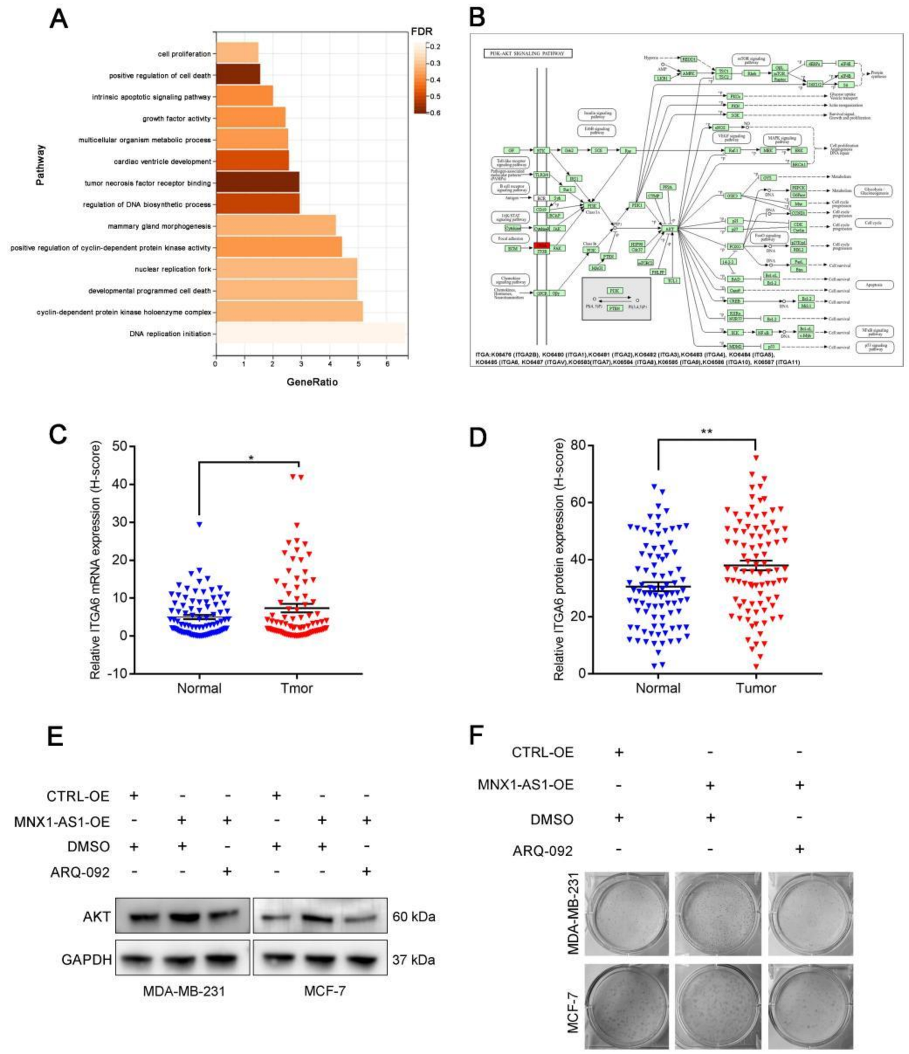

Figure. S2.

(A) GO analysis for all altered genes after knockdown of MNX1-AS1.

(B) Pathway map of PI3K/AKT pathway.

(C) Statistical analysis of ISH expression in BC tissues (n=90) and paired normal tissues (n=90).

(D) Statistical analysis of IHC expression in BC tissues (n=90) and paired normal tissues (n=90).

(E) The AKT inhibitor ARQ-092 reverses the promoting effect of MNX1-AS1 on AKT protein.

(F) The promotion of BC proliferation was partially reversed by ARQ-092.

Data are presented as mean ± SEM. * $p < 0.05$, ** $p < 0.01$

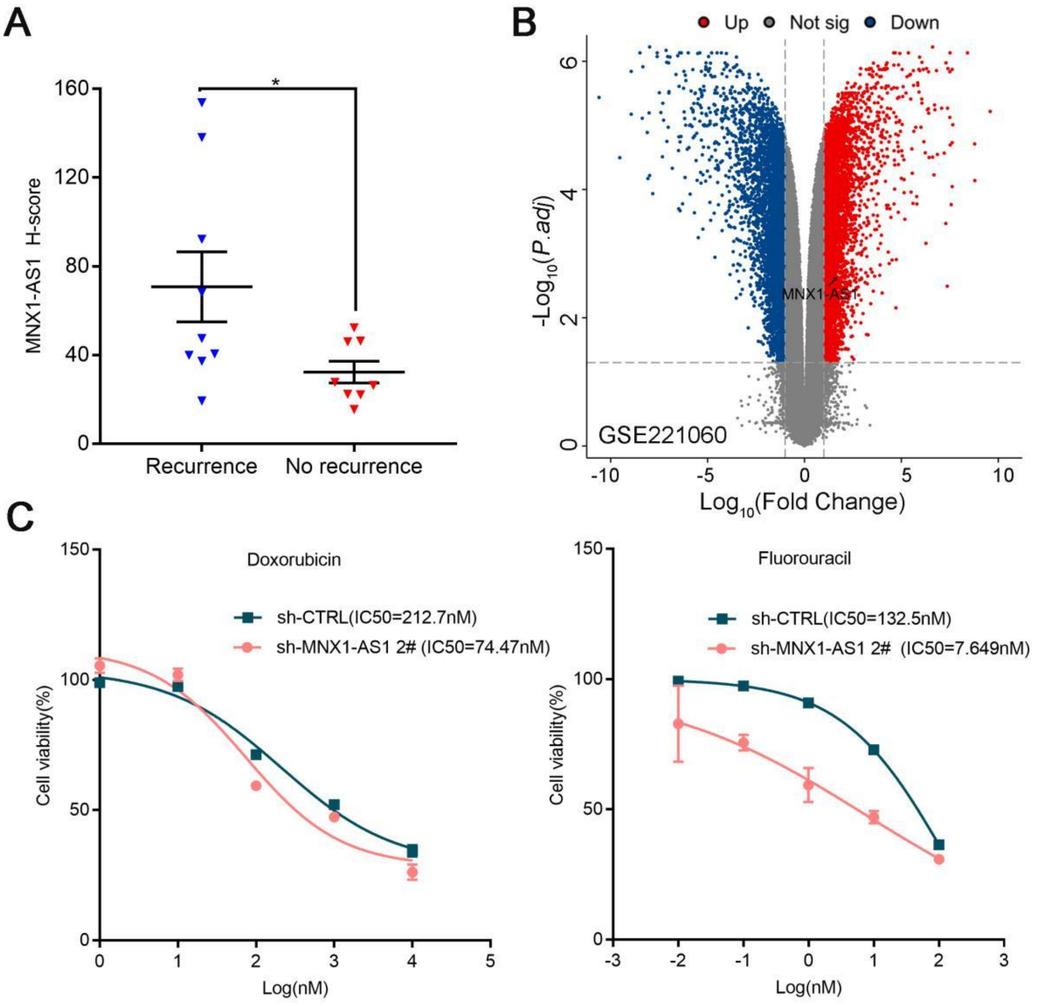

Figure. S3.

(A) FISH expression levels in non-recurrent (n=9) vs recurrent (n=8) breast cancer patients.

(B) The scatter plot of lncRNA expression profiles in chemotherapy-sensitive vs. resistant breast cancer tissues (GSE221060).

(C) Changes in IC50 values of the doxorubicin and 5-fluorouracil on BC cells after MNX1-AS1 knockdown. Data are presented as mean ± SEM. * $p < 0.05$, ** $p < 0.01$ $p < 0.05$, ** $p < 0.01$.

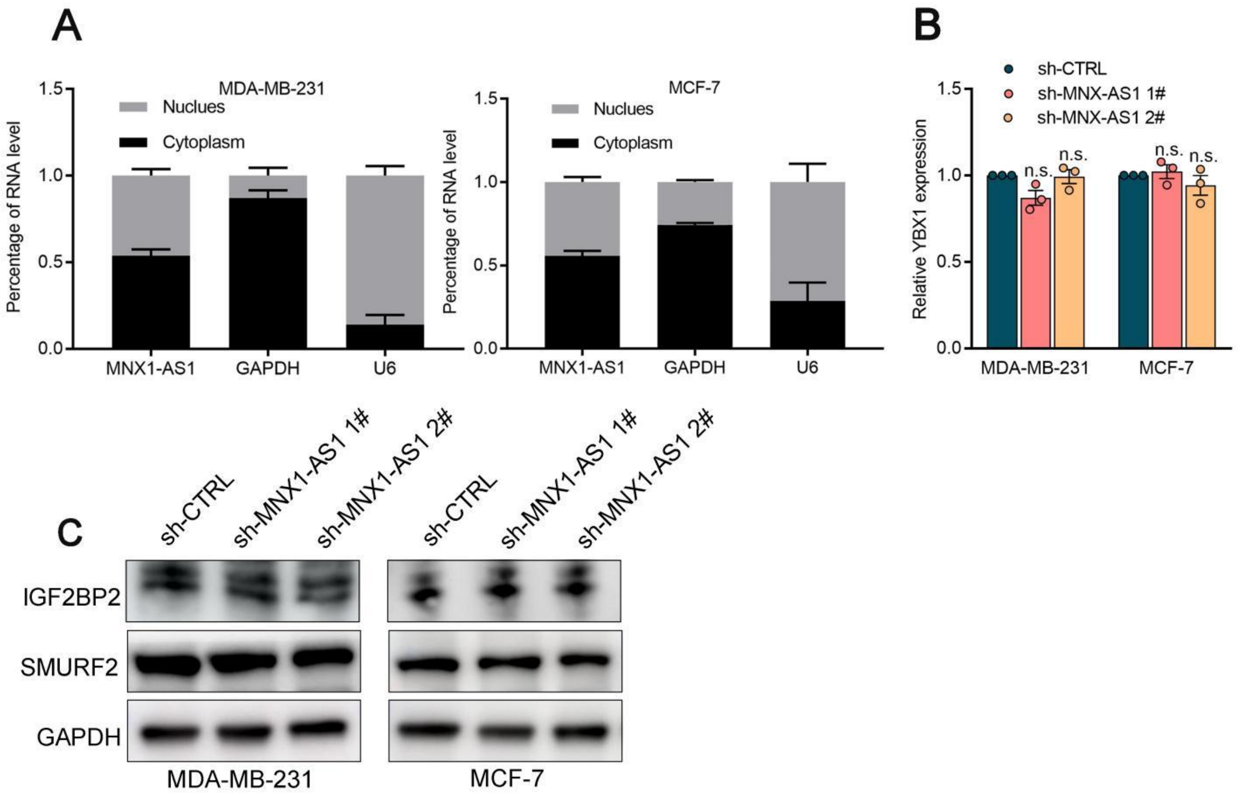

Figure. S4.

(A) The subcellular fractionation assays were used to determine the distribution of lncRNA MNX1-AS1 in MDA-MB-231 and MCF-7 cells.

(B) YBX1 mRNA levels showed no significant changes after MNX1-AS1 knockdown in breast cancer cells.

(C) Western blot analysis confirmed that the knockdown of the MNX1-AS1 gene does not affect the expression of the SMURF2 and IGF2BP2 protein.

Data are presented as mean ± SEM. * $p < 0.05$, ** $p < 0.01$.

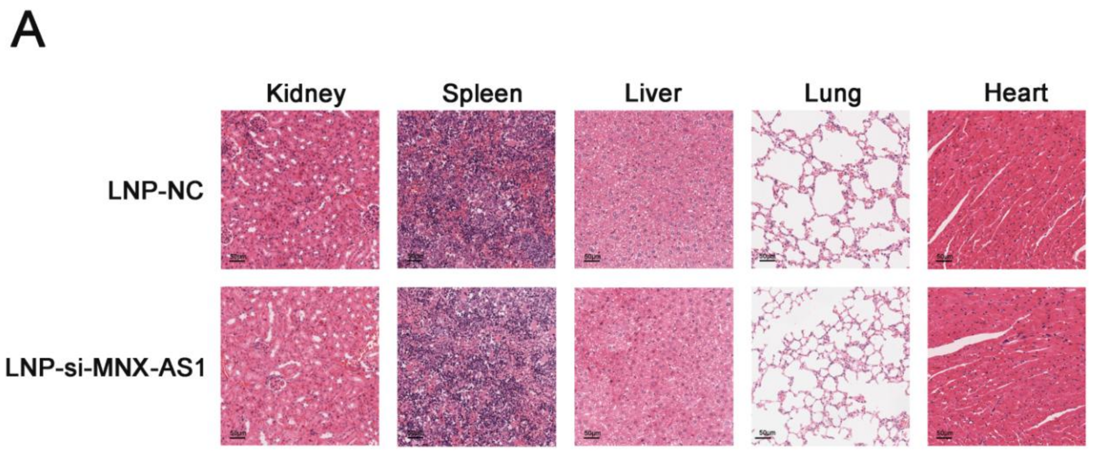

Figure. S5.

(A) Representative H&E staining of major organs including the liver, kidney, lung, spleen, and heart.

Table S1. Prediction of proteins binding to MNX1-AS1 based on starbase database.

RBP	geneName	geneType	clusterNum	clipExpNum	clipIDnum	pancancerNum
ELAVL1	MNX1-AS1	lincRNA	5	1	5	15
EIF4A3	MNX1-AS1	lincRNA	4	2	4	11
NOP58	MNX1-AS1	lincRNA	4	2	4	15
HNRNPA1	MNX1-AS1	lincRNA	3	2	3	14
HNRNPC	MNX1-AS1	lincRNA	3	3	4	14
CSTF2T	MNX1-AS1	lincRNA	2	1	2	16
FBL	MNX1-AS1	lincRNA	2	1	2	19
FUS	MNX1-AS1	lincRNA	2	2	2	16
IGF2BP2	MNX1-AS1	lincRNA	2	1	2	11
RBFOX2	MNX1-AS1	lincRNA	2	1	2	16
TAF15	MNX1-AS1	lincRNA	2	2	2	11
UPF1	MNX1-AS1	lincRNA	2	1	2	10
CNBP	MNX1-AS1	lincRNA	1	1	1	15
FMR1	MNX1-AS1	lincRNA	1	1	1	11
HNRNPK	MNX1-AS1	lincRNA	1	1	1	15
IGF2BP1	MNX1-AS1	lincRNA	1	1	1	13
LIN28	MNX1-AS1	lincRNA	1	1	1	11
MOV10	MNX1-AS1	lincRNA	1	2	2	10
NUMA1	MNX1-AS1	lincRNA	1	1	1	9
PRPF8	MNX1-AS1	lincRNA	1	1	1	9

RANGAP1	MNX1-AS1	lincRNA	1	1	1	15
RBM10	MNX1-AS1	lincRNA	1	1	1	15
RBM5	MNX1-AS1	lincRNA	1	1	1	13
SRSF1	MNX1-AS1	lincRNA	1	1	1	16
U2AF2	MNX1-AS1	lincRNA	1	2	2	12
ZNF184	MNX1-AS1	lincRNA	1	1	1	15

Table S2. Primers for qRT-PCR, siRNAs,shRNAs and the company for antibody.

**Primers used for qRT-PCR**	
GAPDH-F	GCTCTCTGCTCCTCCTGTTC
GAPDH-R	ACGACCAAATCCGTTGACTC
MNX1-AS1-F	AAGGTAGCCACCAAACAC
MNX1-AS1-R	AGACTCACGTAGCACTGT
YBX1-F	CCCCAGGAAGTACCTTCGC
YBX1-R	AGCGTCTATAATGGTTACGGTCT
IGF2BP2-F	AGCTAAGCGGGCATCAGTTTG
IGF2BP2-R	CCGCAGCGGGAAATCAATCT
ITGA6-F	GGCGGTGTTATGTCCTGAGTC
ITGA6-R	AATCGCCCATCACAAAAGCTC
**ChIP primers**	
YBX1-ITGA6-F	TGGGAAGACAGGAATCAATGGTCC
YBX1-ITGA6-R	AAGGGCTCCCTCTGCTTGGA
**siRNAs and shRNA**	
si-YBX1	GGACGGCAATGAAGAAGAT
si-IGF2BP2	CATGCCGCATGATTCTTGA
si-METTL3	GCUCAACAUACCCGUACUATT
sh-MNX1-AS1 1#	AGGUAGCCACCAAACACAUGCAUAA
sh-MNX1-AS1 2#	GAGUCUUGCAAAGAGGAGAUCUUUA
sh-MNX1-AS1 3#	CAUACAACUCGACAGAGUCACAGAA

**Antibody**	**Company**
GAPDH	Proteintech
IgG	Millipore
YBX1	Abcam
SMURF2	Cell Signaling Technology
ITGA6	Proteintech
IGF2BP2	Proteintech
PI3K	Cell Signaling Technology
P-PI3K	Affinity Biosciences
AKT	Cell Signaling Technology
P-AKT	Cell Signaling Technology
**FISH probe sequences**	
LncRNA MNX1-AS1	CAACAGTCTGCAGAGGGCCCTGCGTGAATCT GAAAATGCGGGGCC
